# Inflammatory regulation of glucocorticoid metabolism in mesenchymal stromal cells

**DOI:** 10.1002/art.34414

**Published:** 2012-06-26

**Authors:** Mohammad M Ahasan, Rowan Hardy, Christopher Jones, Kirren Kaur, Dominika Nanus, Maria Juarez, Stuart A Morgan, Zaki Hassan-Smith, Cécile Bénézech, Jorge H Caamaño, Martin Hewison, Gareth Lavery, Elizabeth H Rabbitt, Andrew R Clark, Andrew Filer, Christopher D Buckley, Karim Raza, Paul M Stewart, Mark S Cooper

**Affiliations:** 1 University of Birmingham and Queen Elizabeth HospitalEdgbaston, Birmingham, UK; 2 University of BirminghamEdgbaston, Birmingham, UK; 3 UCLA–Orthopedic HospitalLos Angeles, California; 4 Kennedy Institute of Rheumatology, University of OxfordLondon, UK

## Abstract

**Objective:**

Tissue glucocorticoid (GC) levels are regulated by the GC-activating enzyme 11β-hydroxysteroid dehydrogenase type 1 (11β-HSD1). This enzyme is expressed in cells and tissues arising from mesenchymal stromal cells. Proinflammatory cytokines dramatically increase expression of 11β-HSD1 in stromal cells, an effect that has been implicated in inflammatory arthritis, osteoporosis, obesity, and myopathy. Additionally, GCs act synergistically with proinflammatory cytokines to further increase enzyme expression. The present study was undertaken to investigate the mechanisms underlying this regulation.

**Methods:**

Gene reporter analysis, rapid amplification of complementary DNA ends (RACE), chemical inhibition experiments, and genetic disruption of intracellular signaling pathways in mouse embryonic fibroblasts (MEFs) were used to define the molecular mechanisms underlying the regulation of 11β-HSD1 expression.

**Results:**

Gene reporter, RACE, and chemical inhibitor studies demonstrated that the increase in 11β-HSD1 expression with tumor necrosis factor α (TNFα)/interleukin-1β (IL-1β) occurred via the proximal HSD11B1 gene promoter and depended on NF-κB signaling. These findings were confirmed using MEFs with targeted disruption of NF-κB signaling, in which RelA (p65) deletion prevented TNFα/IL-1β induction of 11β-HSD1. GC treatment did not prevent TNFα-induced NF-κB nuclear translocation. The synergistic enhancement of TNFα-induced 11β-HSD1 expression with GCs was reproduced by specific inhibitors of p38 MAPK. Inhibitor and gene deletion studies indicated that the effects of GCs on p38 MAPK activity occurred primarily through induction of dual-specificity phosphatase 1 expression.

**Conclusion:**

The mechanism by which stromal cell expression of 11β-HSD1 is regulated is novel and distinct from that in other tissues. These findings open new opportunities for development of therapeutic interventions aimed at inhibiting or stimulating local GC levels in cells of mesenchymal stromal lineage during inflammation.

An increase in tissue levels of glucocorticoids (GCs) is an important component of the inflammatory response (1). Impairment of these counterregulatory responses (e.g., by impaired GC synthesis or GC receptor blockage) is associated with high mortality in inflammatory states in humans and animals (2, 3). The antiinflammatory actions of GCs are mediated through inhibition of proinflammatory signaling pathways such as NF-κB, activator protein 1 (AP-1), and MAPKs.

At the tissue level, the action of GCs is regulated by activity of the enzyme 11β-hydroxysteroid dehydrogenase type 1 (11β-HSD1) (4, 5), which interconverts inactive GCs such as cortisone and dehydrocorticosterone with their active counterparts cortisol and corticosterone. Expression of 11β-HSD1 appears to be a common feature in all cell types that have a mesodermal origin (6). Although 11β-HSD1 activity can be bidirectional, in these cells the activity is primarily in the reductase direction (converting inactive GCs to their active form). In osteoblasts, synovial fibroblasts, adipocytes, and myocytes, 11β-HSD1 expression, and consequent GC activation, has been postulated to play a role in the development of inflammation-associated osteoporosis, arthritis, obesity, and myopathy, respectively (7–11). We have previously reported that proinflammatory cytokines such as tumor necrosis factor α (TNFα) and interleukin-1β (IL-1β) increase the expression and activity of 11β-HSD1 in these mesenchymal stromal cell types and tissues (7, 10, 12, 13). In contrast, proinflammatory cytokines have no effect on 11β-HSD1 expression in hepatocytes, monocytes, or lymphocytes (10, 14, 15). Furthermore, combined treatment with GCs and proinflammatory cytokines synergistically increases expression and activity of 11β-HSD1 in osteoblasts, synovial fibroblasts, and myocytes (13).

This ability of GCs to further stimulate, rather than inhibit, inflammation-associated 11β-HSD1 expression in mesenchymal stromal cells may be a feedforward mechanism to selectively increase local GC action in these cells during inflammation (16). The molecular mechanisms involved in regulating expression of 11β-HSD1 in cells such as hepatocytes, monocytes, and lymphocytes have been explored previously (14, 15, 17). The best-characterized of these mechanisms is the increase in 11β-HSD1 expression in hepatocytes in response to GCs; this is mediated by members of the CCAAT/enhancer binding protein family and requires new protein synthesis (17). However, to date none of these studies have characterized signaling systems involved in mediating the effects of proinflammatory cytokines and GCs in mesenchymal stromal cells. This raises the possibility that novel regulatory pathways regulate these effects. Furthermore, the presence of distinct regulatory mechanisms in musculoskeletal cells might enable tissue-specific regulation of 11β-HSD1 activity. In this study we examined the mechanisms underlying the regulation of 11β-HSD1 expression and activity in osteoblasts, synovial fibroblasts, and myoblasts.

## MATERIALS AND METHODS

### Cell and tissue culture

Reagents were obtained from Sigma unless noted otherwise. Primary synovial fibroblasts were generated from synovial tissue obtained at the time of knee arthroplasty from patients with rheumatoid arthritis (RA) according to the American College of Rheumatology 1987 classification criteria (18) or with osteoarthritis (OA), as previously described (12). Additionally, synovial fibroblasts were generated from normal synovial tissue isolated from subjects undergoing knee arthroscopy for noninflammatory conditions. Isolated fibroblasts were grown in RPMI 1640 medium containing 1% (volume/volume) nonessential amino acids, 1% penicillin/streptomycin, 1% sodium pyruvate, 2 m*M* glutamine, and 20% fetal bovine serum (FBS; Labtech). MG-63 osteosarcoma cells were cultured in modified Eagle's medium (MEM) with 10% FBS, 1% nonessential amino acids, and 2 mmoles/liter l-glutamine. Human primary dermal fibroblasts were isolated from human skin by explant culture using the same medium used for primary synovial fibroblasts (9). C2C12 myocytes (European Collection of Cell Cultures) were cultured in high-glucose Dulbecco's MEM (DMEM) with 10% FBS, 1% l-glutamine, and 4.5 gm/liter glucose (PAA). When cells had reached ∼80% confluence, differentiation of myocytes was initiated by replacing existing medium with high-glucose DMEM (5% horse serum, 1% l-glutamine, 4.5 gm/liter glucose). Primary human hepatocytes from a 57-year-old man were obtained via a commercial source (HepAlert) and cultured in Williams E medium (phenol red free), 1% insulin–transferrin–sodium selenite, and 1% l-glutamine. Mouse embryonic fibroblasts (MEFs) from wild-type mice or mice with targeted deletion of RelA (p65), RelB, or dual-specificity phosphatase 1 (DUSP-1) were generated using previously characterized mouse models (19–21). Mouse gastrocnemius muscle tissue was obtained from wild-type C57BL/6 mice (n = 4) immediately postmortem. The tissue was incubated in DMEM with 10% FBS, 1% l-glutamine, and 4.5 gm/liter glucose and used immediately in experiments.

Cells were cultured with proinflammatory cytokines (TNFα/IL-1β [0.1–10 ng/ml]) and GCs (cortisol/dexamethasone [DEX] [1–100 n*M*]) alone or in combination. In some experiments chemical inhibitors of cell signaling pathways were added. Cells were pretreated with inhibitor for 2 hours and then treated with cytokines and GCs for 24 hours. Inhibitors used included p38 MAPK inhibitors (SB202190 [10 μ*M*], SB203580 [15 μ*M*], and SB239063 [10 μ*M*]), ERK inhibitor (PD98059 [15 μ*M*]), protein kinase C inhibitor (Go6976 [7.9 n*M*]), NF-κB inhibitors (parthenolide [10 μ*M*], pyrrolidine dithiocarbamate [PDTC] [5 m*M*], lactacystin [10 μ*M*], and TLCK [20 μ*M*]), AP-1 inhibitor (curcumin [100 n*M*]), and DUSP/MAPK phosphatase inhibitors (sodium orthovanadate [10 μ*M*] and Ro-31-8220 [10 μ*M*]).

All studies were approved by the local research ethics committee, and all subjects provided written informed consent. All animal experiments and procedures were approved by the University of Birmingham Animal Care and Use Committees.

### RNA extraction and reverse transcription (RT)

RNA was extracted from cultured fibroblasts using the single-step extraction method (TRI Reagent). Aliquots (1 μg) of RNA were then reverse transcribed using random hexamers (Promega).

### RNA ligase–mediated rapid amplification of complementary DNA (cDNA) ends (RLM-RACE) analysis of 11β-HSD1 messenger RNA (mRNA) transcripts

Total RNA was isolated from MG-63 cells and synovial fibroblasts (from patients with either OA or RA) that were left untreated or treated with 10 ng/ml IL-1β, 100 nmoles/liter DEX, or a combination of IL-1β and DEX. DNA was removed using a Turbo DNA-free kit according to the instructions of the manufacturer (Ambion). RLM-RACE was carried out using a GeneRacer kit as recommended by the manufacturer (Invitrogen).

For amplification of the 5′ end of the cDNA, the 11β-HSD1 gene–specific primer (5′-GGGCAACAAATTGCTCTGCGAAGGT-3′) and the GeneRacer 5′ primer supplied with the kit were used. After gel purification, PCR products were cloned into pGEM-T Easy vector (Promega) and sequenced.

### Assays for 11β-HSD1 mRNA stability

The stability of 11β-HSD1 mRNA relative to 18S ribosomal RNA (rRNA) was determined using real-time RT–polymerase chain reaction (PCR). MG-63 cells were treated for 4 hours with 10 ng/ml IL-1β, 100 n*M* DEX, or a combination of IL-1β and DEX. Cells were then treated with 1 μg/ml actinomycin D to arrest gene transcription. The change in 11β-HSD1 mRNA expression relative to 18S rRNA was measured after a further 4 hours and 20 hours, and results were used to derive the ΔΔC_t_ value.

### Real-time PCR

Expression of mRNA was assessed by real-time PCR with an ABI 7500 system (Applied Biosytems) using a previously reported technique (13). Reaction conditions were as follows: 50°C for 2 minutes, 95°C for 10 minutes, and 40 cycles of 95°C for 15 seconds and 60°C for 1 minute. Data were obtained as C_t_ values (the cycle number at which logarithmic PCR plots cross a calculated threshold line) and used to determine ΔC_t_ values (C_t_ of target gene − C_t_ of housekeeping gene) as raw data for gene expression (high ΔC_t_ = low gene expression). The fold change in gene expression was determined by subtracting ΔC_t_ values of treated cells from the values obtained with their respective control samples. The resulting ΔΔC_t_ values were then used to calculate fold change in gene expression according to the equation 2^−ΔΔACt^.

### Enzyme assays for 11β-HSD

Confluent cell monolayers and tissue explants were assayed using a thin-layer chromatography–based system as previously reported (22). Cortisone along with tracer amounts of ^3^H-cortisone (generated in-house as previously described [22]) was added, and tissue was incubated at 37°C for various time intervals (2–24 hours) to optimize measurement of cell or tissue activity.

### Examination of 11β-HSD1 gene reporter activity using dual-luciferase reporter assays

Previously described constructs containing 11β-HSD1 promoter sequences immediately 5′ to the classic exon 1 of the human HSD11B1 gene (23) were subcloned into pGL3-Enhancer Vector (Promega) in order to examine the effect of IL-1β or GCs on 11β-HSD1 promoter activity. MG-63 cells were transiently transfected with the promoter constructs using Lipofectamine Plus according to the recommendations of the manufacturer (Invitrogen). Cells were grown to 50–60% confluence in 24-well plates before transfection with 0.8 μg DNA, 8 μl PLUS reagent, 8 μl phRL-TK *Renilla* luciferase vector (0.02 μg; Promega), and 2 μl Lipofectamine reagent made up to a final volume of 250 μl with serum free medium. After treatments were added, cells were incubated for a further 48 hours. All transfections were performed in triplicate. Control wells contained cells in serum-free medium. After incubation for 3 hours, 1 ml of growth medium containing FBS was added per well. Cells were treated and incubated for 48 hours.

Transfected cells were washed in phosphate buffered saline and lysed in 100 μl 1× passive lysis buffer (Promega). A Dual-Luciferase Reporter Assay System (Promega) was used to measure the promoter activity of each construct (in relative light units, corrected for transfection efficiency).

### RelA nuclear translocation assays

The translocation of RelA (p65) from cytoplasm to nucleus was examined in primary synovial fibroblasts that were left untreated or treated with TNFα or with a combination of TNFα and DEX, with or without NF-κB inhibitors (parthenolide and PDTC). Cells were pretreated for 2–17 hours before nuclear isolation. RelA protein content within the nucleus was determined by enzyme-linked immunosorbent assay using a TransAM NF-κB transcription factor assay kit (Active Motif).

### Statistical analysis

The normality of data distribution was examined using the Kolmogorov-Smirnov and Shapiro-Wilk tests. Data are reported as the mean ± SD of replicate mean values. One-way analysis of variance and Student's *t*-tests were performed using SPSS Data Editor.

## RESULTS

### Proinflammatory cytokine induction of 11β-HSD1 does not require new protein synthesis

**Figure 1:**
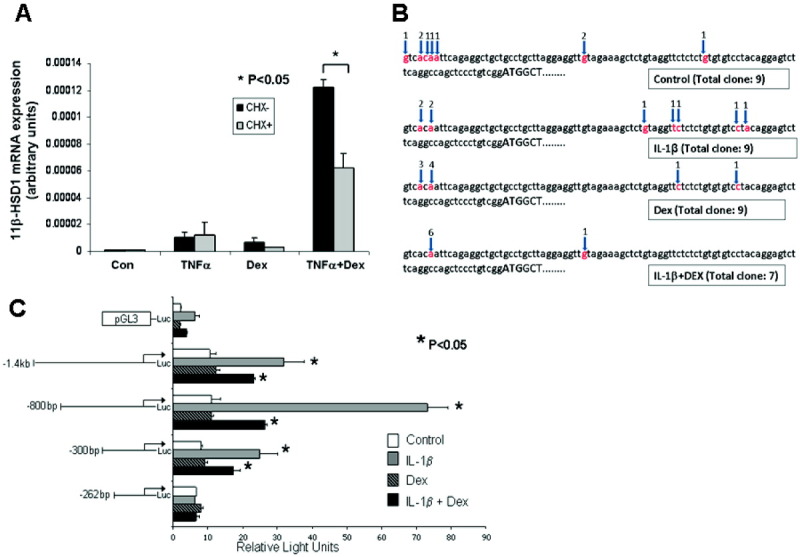
Proinflammatory cytokine and glucocorticoid regulation of 11β-hydroxysteroid dehydrogenase type 1 (11β-HSD1) expression at the promoter level. A, Effect of the protein synthesis inhibitor cycloheximide (CHX) on HSD11B1 gene transcription in rheumatoid arthritis (RA) synovial fibroblasts. CHX did not affect induction of 11β-HSD1 expression by tumor necrosis factor α (TNFα), but partially reduced the additional expression obtained with TNFα and dexamethasone (DEX) combined. Con = control. B, Rapid amplification of complementary DNA ends (RACE) analysis. Experiments were performed using RA synovial fibroblasts, osteoarthritis (OA) synovial fibroblasts, and MG-63 osteoblasts. Data shown are for RA synovial fibroblasts; similar results were obtained with OA synovial fibroblasts and MG-63 osteoblasts (data not shown). RACE analysis demonstrated that RA and OA synovial fibroblasts and MG-63 osteoblasts use the proximal promoter, and there is no difference in transcription start site according to treatment or disease state. Arrows indicate the transcriptional start site for each transcript. C, Results of dual-luciferase activity assays in MG-63 cells. Interleukin-1β (IL-1β) stimulated proximal promoter activity, but no effect was seen with DEX alone. The combination of IL-1β and DEX also stimulated promoter activity, but not to a greater extent than that obtained with IL-1β alone. *P* values are versus control. Values in A and C are the mean ± SD.

Primary synovial fibroblasts from RA patients were treated with TNFα and/or DEX, in the presence or absence of cycloheximide. Analysis of the induction of 11β-HSD1 demonstrated that the increase in 11β-HSD1 expression did not depend on new protein synthesis (Figure 1A). In contrast, the synergistic increase in 11β-HSD1 expression with combined TNFα and GCs was partially inhibited by cycloheximide. This indicated that the augmenting effect of GCs on TNFα-induced 11β-HSD1 expression is mediated by a mechanism distinct from that of TNFα alone, which is dependent on new protein synthesis. Similar results were obtained in MG-63 cells and C2C12 myoblasts (data not shown).

### Regulation of 11β-HSD1 expression involves the classic gene promoter as demonstrated by 5′-RACE analysis

We examined whether differences in expression of 11β-HSD1 with proinflammatory cytokine and/or GC treatment could arise from differences in promoter usage. Using primary human synovial fibroblasts from patients with RA or OA and MG-63 cells, 5′-RACE analysis of mRNA transcripts demonstrated minor variations in the 5′ end of the mRNA transcript, but all transcripts originated from usage of the conventional proximal promoter (Figure 1B and data not shown). Furthermore, there was no difference in the origin or length of the transcripts with IL-1β or DEX treatment. Additionally, there was no difference in transcript length or origin between synovial fibroblasts obtained from patients with RA and those from patients with OA (data not shown).

Possible mechanisms for the regulation of 11β-HSD1 mRNA expression with combination GC plus IL-1β treatment were explored. In MG-63 cells, GCs did not affect the half-life of 11β-HSD1 mRNA expression in the presence of actinomycin D (an inhibitor of mRNA transcription), as measured by real-time PCR (half-life 12.5 hours with IL-1β and 13 hours with IL-1β plus DEX; *P* not significant) (data available from the corresponding author upon request).

### Regulation of 11β-HSD1 expression with proinflammatory cytokines occurs via the proximal promoter of HSD11B1

To determine whether induction of 11β-HSD1 expression by proinflammatory cytokines and/or GCs was due to activation of specific regions of the gene promoter for this enzyme, promoter-reporter assays were carried out in MG-63 cells, using dual-luciferase reporter constructs containing different fragments of the HSD11B1 proximal gene promoter (Figure 1C). Treatment with IL-1β increased promoter activity of the 1.4-kb sequence upstream of the HSD11B1 gene, in a dose-dependent manner. Analysis using previously published constructs of various lengths (23) demonstrated that IL-1β treatment stimulated promoter activity with all fragments greater than >300 bp in length, but this was not observed with the 262-bp construct (Figure 1C). Additional reporter activity was found in the 800-bp fragment, compared to the 1.4-kb sequence. This indicated that the IL-1β signal is mediated through several distinct regions of the proximal promoter. Importantly, in contrast to the findings with regard to mRNA expression and enzyme activity, DEX treatment did not induce reporter activity, and treatment with DEX and IL-1β in combination led to reduced gene reporter activity compared to that observed with IL-1β alone. This demonstrated an inhibitory effect of DEX on induction of 11β-HSD1 expression by IL-1β in this region of the promoter.

### Suppression of 11β-HSD1 expression by chemical inhibitors of NF-κB signaling

**Figure 2:**
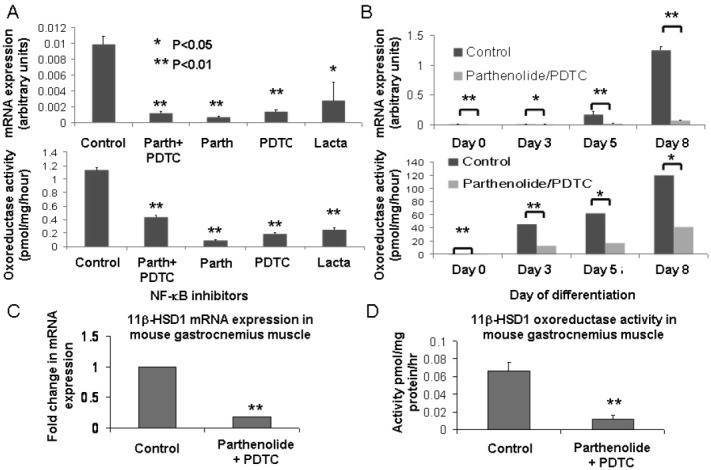
Effect of NF-κB inhibitors on 11β-hydroxysteroid dehydrogenase type 1 (11β-HSD1) oxoreductase expression and activity in C2C12 myoblasts. The inhibitors used were parthenolide (Parth) (10 μ*M*), pyrrolidine dithiocarbamate (PDTC) (5 m*M*), and lactacystin (Lacta) (10 μ*M*). A and B, Effects of NF-κB inhibition on basal expression and activity of 11β-HSD1 (A) and on the increase seen during myoblast differentiation (B). C and D, Effects of NF-κB inhibition on 11β-HSD1 expression (C) and activity (D) in freshly isolated muscle tissue from 4 mice. Values are the mean ± SD. *P* values in A, C, and D are versus control.

The underlying mechanism for regulation of 11β-HSD1 expression was examined initially in myocytes, using a range of chemical inhibitors with selectivity for various signaling pathways. Inhibitors of AP-1, ERK, and protein kinase C signaling had no effect on either basal expression of 11β-HSD1 or induction of expression of the enzyme in response to proinflammatory cytokines or GCs (data not shown). In contrast, a range of inhibitors of NF-κB signaling had a significant inhibitory effect on basal 11β-HSD1 mRNA expression and enzyme activity in C2C12 myoblasts (Figure 2A). In a model of differentiation from a pluripotential mesenchymal cell toward myotubes, NF-κB inhibition (using parthenolide plus PDTC) was effective in inhibiting 11β-HSD1 activity at all stages of differentiation (Figure 2B). This inhibition was rapidly reversible upon withdrawal of the inhibitor (data available from the corresponding author upon request).

To ensure that NF-κB activity was the prime regulator of 11β-HSD1 activity in musculoskeletal tissue ex vivo as well as in vitro, we examined the effect of NF-κB inhibition on mouse muscle tissue collected immediately postmortem (Figures 2C and D). Expression of 11β-HSD1 in this tissue was stable over 18 hours of incubation. Inhibition of NF-κB using parthenolide plus PDTC resulted in a rapid and substantial decrease in 11β-HSD1 mRNA expression and enzyme activity in this tissue.

### Suppression of cytokine-induced 11β-HSD1 expression by chemical inhibitors of the NF-κB pathway

**Figure 3:**
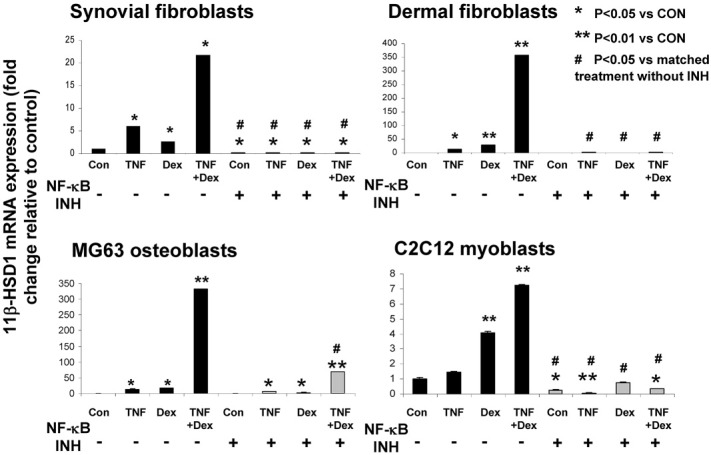
NF-κB is the major mediator of the induction of 11β-HSD1 by TNFα in fibroblasts (normal synovial and dermal; n = 3 independent patient lines), osteoblasts, and myoblasts. The effect of NF-κB inhibition (INH) with parthenolide (10 μ*M*) plus pyrrolidine dithiocarbamate (5 m*M*) on the induction of 11β-HSD1 activity by TNFα, DEX, and their combination is shown. Values are the mean ± SD. See Figure 1 for other definitions.

The effect of NF-κB inhibitors on the increased expression of 11β-HSD1 following treatment with proinflammatory cytokines and/or GCs was examined in a range of cells arising from the mesenchymal stromal cell lineage (Figure 3). NF-κB inhibition using parthenolide and PDTC was highly effective in attenuating 11β-HSD1 activity induced by TNFα or by combined TNFα and GC. To ensure that the responses observed in synovial fibroblasts were representative of changes in normal synovial fibroblasts, synovial fibroblasts were obtained from healthy donors. Responses obtained using synovial fibroblasts from patients with RA were similar to those of normal fibroblasts (data not shown). In contrast, 11β-HSD1 activity in primary human hepatocytes was not responsive to proinflammatory cytokines and was not affected by NF-κB inhibition (data available from the corresponding author upon request).

### The increase in 11β-HSD1 expression with proinflammatory cytokines is mediated through RelA (p65) activity

**Figure 4:**
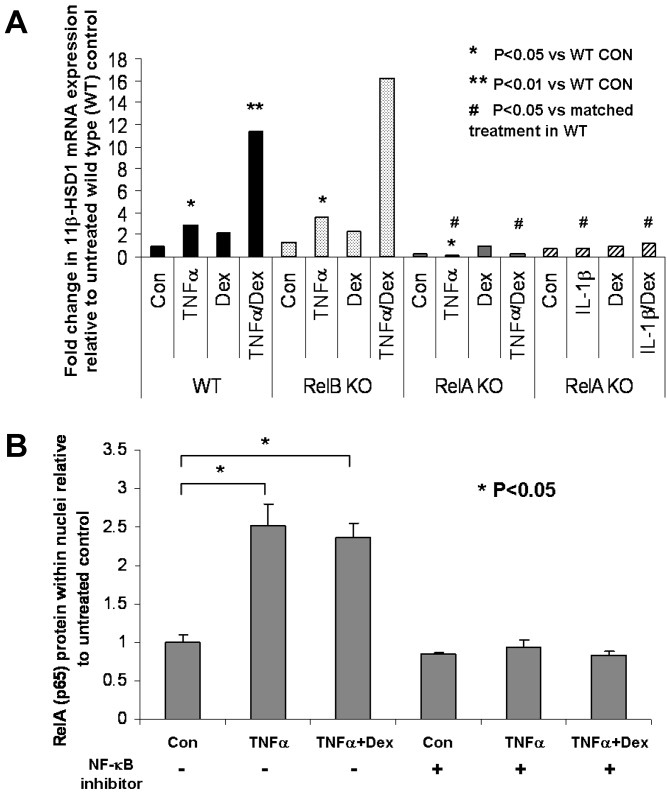
Molecular analysis of the role of NF-κB in regulation of 11β-HSD1 expression. A, Effect of treatments (TNFα, IL-1β, DEX, combined TNFα and DEX, or combined IL-1β and DEX) on 11β-HSD1 mRNA expression in mouse embryonic fibroblasts from wild-type (WT), RelB-knockout (KO), and RelA-knockout mice (all on a C57BL/6 background; lines from 9 embryos). B, Effect of TNFα with or without DEX and with or without NF-κB inhibitor (INH) on RelA nuclear translocation in RA synovial fibroblasts. RelA (p65) translocated to the nucleus in response to TNFα treatment. This effect was not blocked by glucocorticoid, but was completely inhibited by NF-κB inhibitor. Values are the mean ± SD. See Figure 1 for other definitions.

To further define the specific components of NF-κB signaling that regulate 11β-HSD1 expression, we examined the effect of targeted deletion of components of both the classic and the alternate NF-κB signaling cascade. Deletion of RelB in MEFs had no impact on the levels of 11β-HSD1 observed after treatment with proinflammatory cytokine or GC (Figure 4A). In contrast, RelA deletion entirely prevented the induction of 11β-HSD1 in response to TNFα or IL-1β (Figure 4A shows mRNA expression data; these changes were paralleled by changes in enzyme activity [data not shown]). These results indicate that the classic NF-κB pathway has a central role in mediating cytokine induction of 11β-HSD1.

### Glucocorticoids do not prevent TNFα-induced translocation of RelA to the nucleus

GCs have been reported to have a significant inhibitory effect on proinflammatory cytokine–induced nuclear translocation of NF-κB in some cells and tissues (24–26). We therefore examined the effect of GC treatment on cytokine-induced translocation of RelA from cytoplasm to nucleus in synovial fibroblasts obtained from RA patients. Treatment with TNFα increased the level of RelA protein in the nucleus (Figure 4B). This effect was efficiently blocked by an NF-κB inhibitor. In contrast, GCs had no impact on TNFα-stimulated RelA translocation, indicating failure to inhibit NF-κB activity at the level of nuclear translocation.

### The p38 MAPK pathway also regulates the effect of proinflammatory cytokines on 11β-HSD1 expression

**Figure 5:**
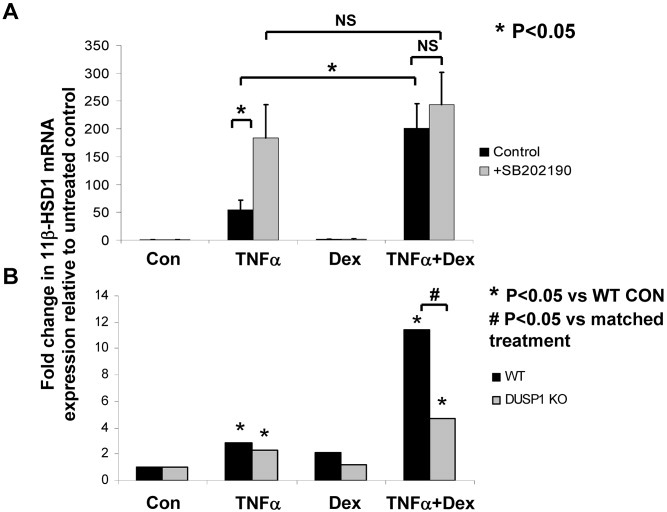
Molecular analysis of the role of p38 MAPK signaling in 11β-HSD1 expression. A, Effect of the p38 MAPK inhibitor SB202190 on the induction of 11β-HSD1. Expression of 11β-HSD1 expression with TNFα plus SB202190 treatment was increased to a similar extent as was observed with TNFα plus DEX treatment. B, Effect of dual-specificity phosphatase 1 (DUSP-1) deletion in mouse embryonic fibroblasts (lines from 4 embryos). The results indicate that inhibition of p38 MAPK by DEX occurs primarily through induction of DUSP-1. Values are the mean ± SD. NS = not significant; WT = wild-type; KO = knockout (see Figure 1 for other definitions).

Proinflammatory cytokines are powerful stimulators, and GCs powerful inhibitors, of the p38 MAPK pathway in stromal cells. We therefore examined whether p38 MAPK signaling was involved in the regulation of 11β-HSD1 activity in response to proinflammatory cytokines or GCs. We found that selective inhibitors of p38 MAPK signaling augmented TNFα stimulation of 11β-HSD1 expression, but had no effect on basal expression (Figure 5A). The increase in 11β-HSD1 expression over that obtained with TNFα treatment alone was equivalent when TNFα was combined with p38 MAPK inhibitor or with DEX. Furthermore, the level of 11β-HSD1 expression after treatment with a combination of TNFα, DEX, and p38 MAPK inhibitor was not significantly different from that observed after treatment with TNFα and DEX without p38 MAPK inhibitor or TNFα and p38 MAPK inhibitor without DEX (Figure 5A). These data indicate that GCs augment cytokine induction of 11β-HSD1 expression via their ability to inhibit p38 MAPK.

### GCs increase the effect of proinflammatory cytokines on 11β-HSD1 expression indirectly through induction of DUSP-1 activity

The finding that the effect of GCs on cytokine-induced expression of 11β-HSD1 is dependent on new protein synthesis (Figure 1A) indicates that this particular effect of GCs is mediated in an indirect manner. In osteoblasts and fibroblasts, GCs primarily inhibit p38 MAPK activity through the induction of DUSP-1 expression and activity (27, 28). Nonspecific chemical inhibitors of DUSP-1 (e.g., sodium orthovanadate; Ro-31-8220) had no effect on TNFα/IL1β induction of 11β-HSD1 in MG-63 cells, but prevented the augmentation of cytokine-induced 11β-HSD1 expression with GCs (data available from the corresponding author upon request). The specific role of DUSP-1 was confirmed in experiments using MEFs from mice with transgenic disruption of DUSP-1 (Figure 5B). Basal and TNFα-stimulated levels of 11β-HSD1 expression were the same in DUSP-1–knockout and wild-type MEFs, but after combined treatment with TNFα and GCs, expression of 11β-HSD1 was significantly reduced in DUSP-1–knockout MEFs.

## DISCUSSION

**Figure 6:**
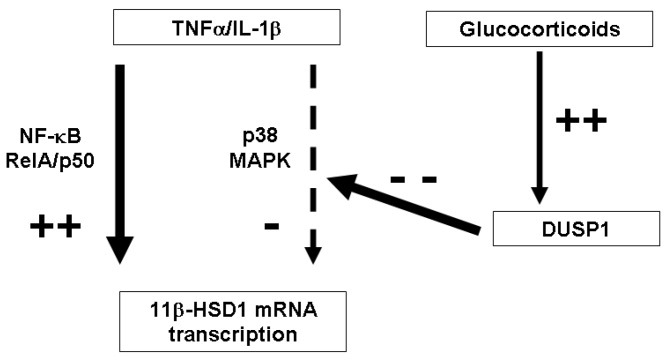
Schematic representation of the pathways by which TNFα/IL-1β and glucocorticoids regulate 11β-HSD1 expression and activity in musculoskeletal cells. TNFα/IL-1β exert both stimulatory and inhibitory effects via NF-κB and p38 MAPK pathways, respectively. The inhibitory pathway can be blocked by glucocorticoids, primarily through a dual-specificity phosphatase 1 (DUSP-1)–dependent mechanism. See Figure 1 for other definitions.

We have previously shown that increased expression of 11β-HSD1 during inflammation is an important mechanism regulating the immune response (12, 13). This increase in expression is common to cells that arise from mesenchymal stromal cells, such as fibroblasts, myoblasts, and osteoblasts (7, 8, 10, 12). Additionally, a synergistic induction of 11β-HSD1 expression by proinflammatory cytokines and GCs is seen in these cell types (13). This synergistic interaction has a significant impact in reducing the production of IL-6 by synovial fibroblasts. In the present study we demonstrated the molecular basis for regulation of 11β-HSD1 in these cells. This regulatory mechanism depends on the relative balance of NF-κB and p38 MAPK signaling pathways (illustrated schematically in Figure 6) and is distinct from mechanisms that regulate 11β-HSD1 activity in other cell types (such as hepatocytes). This feedforward mechanism allows for sustained GC production only in the presence of continuing proinflammatory stimulation.

An unusual finding with regard to the synergistic induction of 11β-HSD1 activity was that GCs augmented proinflammatory signaling, rather than inhibiting it. Our data indicate that this augmentation is due to inhibition of p38 MAPK activity. Another unusual feature is the antiinflammatory role of NF-κB. NF-κB signaling was previously thought to be exclusively proinflammatory, but there are now examples of situations in which it appears to have an important antiinflammatory role (29). The involvement of NF-κB in the production of GCs is also interesting, and at first glance unexpected, since NF-κB signaling is a major target of GCs in many cell types (24). GCs inhibit NF-κB signaling in immune cells and tissues, e.g., T lymphocytes, thymocytes, splenocytes, and lymph nodes (25, 26), but only recently has it been explored in cells of the mesenchymal stromal lineage. For example, GCs are unable to repress NF-κB action in osteoblasts (30). Similar findings in synovial fibroblasts have also been reported (31). Data presented here suggest that this lack of inhibition of NF-κB by GCs is a critical feature of the regulation of 11β-HSD1. This mechanism of regulation ensures that the production of GCs within cells derived from mesenchymal precursors continues to be sustained in the presence of continuing activation of NF-κB signaling, but will decrease rapidly when this signal is removed.

We have demonstrated earlier that during synovial inflammation, the level of active GCs within the joint increases (8). This appears to be a consequence of increased expression of 11β-HSD1 within synovial fibroblasts, and is likely to be an adaptive response to reduce joint inflammation. In many situations it is likely that the increase in GCs will facilitate resolution of the inflammation. The increased expression of 11β-HSD1 within synovial tissue will also amplify the effect of therapeutic GCs since the enzyme also converts inactive prednisone to active prednisolone (32). This will result in the preferential accumulation of active prednisolone within the synovium. However, with chronic inflammation the high levels of GCs within the tissue could adversely affect adjacent bone tissue and undermine the integrity of the joint. Similar considerations apply to inflamed muscle tissue, where short-term increases in GC levels are likely to have a beneficial antiinflammatory effect whereas chronic excess of GC is known to induce muscle atrophy.

The identification of a distinct pathway regulating 11β-HSD1 activity in osteoblasts, synovial fibroblasts, and myoblasts creates new opportunities for therapeutic regulation of 11β-HSD1 activity in these tissues. Therapeutic modulation could be achieved though targeting of either the p38 MAPK or the NF-κB pathway. The demonstration that p38 MAPK inhibitors can increase 11β-HSD1 expression selectively in cells exposed to proinflammatory cytokines suggests that similar drugs could be used to increase GC levels in a tissue- and inflammation-specific manner. The efficacy of p38 MAPK inhibitors in reducing joint inflammation has been demonstrated in experimental models of inflammatory arthritis (33), and it is possible that the mechanism described herein could contribute to this efficacy.

In contrast to strategies designed to increase 11β-HSD1 activity, pharmaceutical companies have focused on developing inhibitors of this enzyme, primarily for use in metabolic disorders associated with GC excess (34). Systemic inhibitors of the 11β-HSD1 enzyme itself are becoming available, but their use in inflammatory states could be complicated by the reported expression of 11β-HSD1 in immune tissue (5, 35). There is a possibility that inhibition of 11β-HSD1 within immune tissue could lead to an exaggerated immune response. Furthermore, 11β-HSD1 activity in the liver is believed to contribute significantly to total-body GC metabolism and its inhibition could have a detrimental impact on the circulating level of GCs, again leading to an increase in the proinflammatory aspects of the immune response. Characterization of the NF-κB pathway as an important regulator of GC synthesis in mesenchymal stromal cells suggests that NF-κB inhibitors could usefully inhibit GC synthesis at sites such as muscle and bone. Rather than promoting an inflammatory response through effects on 11β-HSD1 inhibition in immune cells at sites of chronic inflammation, it is more likely that NF-κB inhibitors would have an antiinflammatory effect, given their widely acknowledged antiinflammatory action on immune cells of hematopoietic origin. Further work should clarify the actual role of targeted inhibition of 11β-HSD1 activity in these situations.

In the present report we describe the molecular regulation of 11β-HSD1 activity in mesenchymal stromal cells. The involvement of two major pathways, normally considered proinflammatory, to reciprocally regulate expression of an antiinflammatory gene suggests that these mechanisms are of fundamental importance in the tissue response to inflammation.

## AUTHOR CONTRIBUTIONS

All authors were involved in drafting the article or revising it critically for important intellectual content, and all authors approved the final version to be published. Dr. Cooper had full access to all of the data in the study and takes responsibility for the integrity of the data and the accuracy of the data analysis.

**Study conception and design.** Ahasan, Hardy, Jones, Kaur, Hewison, Filer, Buckley, Raza, Stewart, Cooper.

**Acquisition of data.** Ahasan, Hardy, Jones, Kaur, Nanus, Juarez, Morgan, Hassan-Smith, Bénézech, Caamaño, Hewison, Lavery, Clark, Filer, Stewart, Cooper.

**Analysis and interpretation of data.** Ahasan, Hardy, Jones, Kaur, Nanus, Morgan, Bénézech, Hewison, Lavery, Rabbitt, Filer, Buckley, Raza, Stewart, Cooper.
